# Evaluation of the Synergistic Activity of Antimicrobial Peptidomimetics or Colistin Sulphate with Conventional Antifungals Against Yeasts of Medical Importance

**DOI:** 10.3390/jof11050370

**Published:** 2025-05-12

**Authors:** Shyam Kumar Mishra, Rajesh Kuppusamy, Christina Nguyen, Jennifer Doeur, Harleen Atwal, Samuel Attard, Kristian Sørensen, Jennifer S. Lin, Edgar H. H. Wong, Alex Hui, Annelise E. Barron, Naresh Kumar, Mark Willcox

**Affiliations:** 1 School of Optometry and Vision Science, Faculty of Medicine and Health, University of New South Wales, Sydney, NSW 2052, Australia; rajesh.kuppusamy@whiteley.com.au (R.K.); christina.nguyen3011@gmail.com (C.N.); jenn.doeur@gmail.com (J.D.); harleenatwal01@gmail.com (H.A.); alex.hui@unsw.edu.au (A.H.); m.willcox@unsw.edu.au (M.W.); 2 Department of Microbiology, Maharajgunj Medical Campus, Institute of Medicine, Tribhuvan University, Kathmandu 44600, Nepal; 3 School of Chemistry, Faculty of Science, University of New South Wales, Sydney, NSW 2052, Australia; s.attard@unsw.edu.au (S.A.); n.kumar@unsw.edu.au (N.K.); 4 Department of Bioengineering, School of Medicine and School of Engineering, Stanford University, Stanford, CA 94305, USA; ksoren@stanford.edu (K.S.); jlin3@stanford.edu (J.S.L.); aebarron@stanford.edu (A.E.B.); 5 School of Chemical Engineering, Faculty of Engineering, University of New South Wales, Sydney, NSW 2052, Australia; edgar.wong@unsw.edu.au; 6 School of Optometry & Vision Science, University of Waterloo, Waterloo, ON N2L 3G1, Canada

**Keywords:** antifungals, antimicrobial peptides, *Candida*, colistin, *Kluyveromyes marxianus*, *Meyerozyma guilliermondii*, *Nakaseomyces glabratus*, peptidomimetics, *Pichia kudriavzevii*, synergy

## Abstract

With rising multidrug-resistant yeast pathogens, conventional antifungals are becoming less effective, urging the need for adjuvants that enhance their activity at lower doses. This study evaluated the synergistic activity of antimicrobial peptidomimetics (TM8 and RK758) or colistin sulphate in combination with conventional antifungals against *Candida albicans*, *C. tropicalis*, *C. parapsilosis*, *Meyerozyma guilliermondii*, *Nakaseomyces glabratus*, *Pichia kudriavzevii* and *Kluyveromyces marxianus*, and *Candidozyma auris* using the checkerboard microdilution test. RK758 was synergistic with fluconazole in 78% of isolates, with the remaining 22% of isolates still showing partial synergy; it showed synergy with amphotericin B in 56% of isolates, and with caspofungin, 78% of isolates exhibited either synergy or partial synergy. TM8 showed synergy with fluconazole in 44% (with partial synergy in another 44%) of isolates, with amphotericin B in 67% of isolates, and with caspofungin in 44% (with partial synergy in another 44%) of isolates. Colistin with fluconazole or caspofungin exhibited synergy or partial synergy in 56% of the isolates. No antagonism was observed in any of the combinations. Additionally, a time-kill assay further demonstrated synergistic activity between fluconazole and TM8 or RK758. The effects of these peptidomimetics on cell membrane integrity were demonstrated in an ergosterol binding assay, supported by SYTOX Green and cellular leakage assays, both indicating a lytic effect. These results suggest that peptidomimetics can synergise with conventional antifungals, offering a potential strategy for combination therapy against yeast infections. The membrane lytic activity of the peptidomimetics likely plays a role in their synergistic interaction with antifungals, thereby enhancing the antimicrobial activities of both compounds at sub-MIC levels.

## 1. Introduction

In most healthy individuals, *Candida* spp. exist as a part of the normal microbiota, particularly on the skin, in the oral cavity, in the gastrointestinal tract, and on the genitals [1]. However, immunocompromised patients face a higher risk of opportunistic infections from these commensal organisms, which range from superficial mucocutaneous presentations to life-threatening systemic manifestations [2]. The rise of multidrug-resistant (MDR) *Candida* spp. clinically poses a formidable burden to healthcare systems worldwide. These pathogens rank among the top 10 most common intensive care unit (ICU) isolates [3] and establish candidaemia as one of the top five nosocomial bloodstream infections [4]. Over the past decade, the annual global incidence of candidaemia has risen from 400,000 to 626,081 cases [5]. However, the majority of *C. albicans* and non-albicans *Candida* spp. originate from different non-sterile sites including the oral cavity, vagina, skin, and urethral orifice [6].

There are more than 150 species of *Candida*, with five accounting for approximately 90% of candidaemia cases: *C. albicans*, *C. glabrata*, *C. tropicalis*, *C. parapsilosis*, and *C. krusei* [7]. These pathogens have been listed as global priority fungal pathogens by the World Health Organization (WHO), highlighting the urgent need for new treatment alternatives beyond the current antifungal options [3]. Recent taxonomic changes have led to the reclassification of several of these *Candida* spp., including *Nakaseomyces glabratus* (formerly *C. glabrata*), *Pichia kudriavzevii* (formerly *C. krusei*), *Meyerozyma guilliermondii* (formerly *C. guilliermondii*), *Candidozyma auris* (formerly *C. auris*), and *Kluyveromyes marxianus* (formerly *C. kefyr*) [8,9]. 

Among these pathogenic yeasts, *C. albicans* is most commonly associated with clinical infections leading to mortality rates ranging between 20% and 50% [10]. *C. albicans* isolates from non-sterile sites in middle-income countries have been reported to exhibit a high prevalence of azole resistance (20–60%) [10]. *P. kudriavzevii* is often associated with infections in individuals with haematological malignancies and transplant recipients [11] and exhibits fast evolution and persistence in hospital environments, causing bloodstream infections, with a mortality rate of up to 67% [12]. *C. auris* is a major emerging antimicrobial-resistant yeast of significant concern [13]. This pathogen is strongly associated with nosocomial infections, with mortality rates of up to 66%, and represents a global health threat [14]. Infections caused by *N. glabratus* are generally endogenous, but nosocomial transmission via healthcare personnel and fomites has also been reported [15]. Similarly, *M. guilliermondii* is an emerging nosocomial opportunistic pathogen associated with antifungal-resistant deep-seated infections [16]. A higher prevalence of fluconazole-resistant *C. parapsilosis* and *C. tropicalis* was noted during COVID-19 as compared to the preceding 2-year period in a surveillance program monitoring invasive fungal infections in 48 hospitals worldwide [17]. 

Generally, polyene monotherapy is not recommended due to its associated high toxicity. Furthermore, studies have shown amphotericin B (AmB) can lead to nephrotoxicity resulting in kidney injury in 50% of patients [18]. Early fungal intervention can reduce fungal load with improvement in patient outcomes. However, invasive fungal infections remain exceptionally difficult to treat due to the limited availability of effective antifungal drugs, their high toxicity, and the emergence of antifungal resistance [19]. Echinocandins, including caspofungin, are the recommended therapy for candidaemia and invasive candidiasis (excluding central nervous system and ocular candidal infections) [20]; however, resistance to these antifungals is not uncommon. 

The current treatment for candidiasis involves antifungal monotherapy with azoles, echinocandins, and polyenes, each targeting different cellular processes to inhibit or kill pathogenic yeast cells [21]. However, the effectiveness of these therapies is increasingly limited by the development of resistant strains, driven by powerful virulence factors such as biofilm formation and phenotypic plasticity. A one-size-fits-all approach is not applicable to antifungal treatment, as each patient’s mycosis requires tailored management [22]. Given the high mortality rate of invasive candidiasis, the increasing population of immunocompromised individuals, and the emergence or reemergence of fungal pathogens, it is crucial to identify effective therapeutics for these yeast pathogens. 

Cationic antimicrobial peptides (AMPs) and their mimics serve as effective alternative antimicrobial agents against multiple fungal pathogens [23]. AMPs are naturally occurring molecules found in microorganisms, plants, and animals, including mammals. AMPs have many positive attributes, but their high synthesis cost, short in vivo lifespan due to susceptibility to proteolysis, and potential toxicity have led to an emphasis on antimicrobial peptidomimetics [24]. Peptidomimetics are synthetic molecules designed to mimic the structure and function of AMPs [25]. They overcome the limitations of natural peptides through their improved stability and potency, alongside their natural antimicrobial properties [26,27,28]. Using lower concentrations of antifungals in synergistic multi-drug combinations may provide reduced drug toxicity and enhanced efficacy through the simultaneous targeting of multiple cellular processes, potentially reducing the likelihood of drug resistance [29]. Recent studies have shown that conventional antifungals used in conjunction with colistin, also known as polymyxin E, a cationic peptide antibiotic, can result in synergy and enhanced antifungal potency against *Candida* spp. [4]. Moreover, this combination reduces the MICs of both the conventional antifungals and colistin, potentially minimising drug toxicity and lowering the risk of resistance associated with higher drug concentrations [30].

This study included two peptidomimetics, TM8 and RK758. TM8 is a C10-terminated heptamer lipopeptoid that forms ellipsoidal micelles and releases monomers from its aggregates during antimicrobial activity [31,32]. While there is little information on the mechanism of action of TM8 alone, a recent study demonstrated its activity on the cell membrane of *C. auris* clade II [33]. This current study expands the mechanistic findings by documenting the cell membrane as the target of this peptoid in other yeast species, including *C. albicans*, *C. tropicalis*, *C. parapsilosis*, *P. kudriavzevii*, *K. marxianus*, *N. glabratus*, *M. guilliermondii*, and *C. auris* clade III. RK758, a short guanidine-functionalised anthranilamide peptidomimetic, is efficacious against antimicrobial-resistant strains, offering a potential pathway for antifungal combination therapy [25]. As RK758 does not need to be internalised by the cell to exert its effects, it may overcome multiple antimicrobial resistance mechanisms [25]. 

It can be postulated that combining peptidomimetics with existing conventional antifungal drugs could enhance antifungal efficacy, accelerate fungal load reduction, and shorten treatment periods. Therefore, this was an empirical screening strategy with a primary objective of evaluating the potential synergistic activity between conventional antifungals and antimicrobial peptidomimetics or colistin sulphate against drug-resistant yeast pathogens. Further, we aimed to evaluate mechanistic insights of peptidomimetics against clinically relevant yeasts. 

## 2. Materials and Methods

### 2.1. Fungal Isolates and Media

Six different genera of yeast cells including *Candida* spp. (*C. tropicalis*, *C. parapsilosis*, and two *C. albicans* isolates), *M. guilliermondii*, *N. glabratus*, *P. kudriavzevii* and *K. marxianus*, and *C. auris* were included in this study, as presented in Table 1. The fungal strains were recovered from stocks in the microbiology laboratory of the School of Optometry and Vision Science, UNSW Sydney. RPMI (Roswell Park Memorial Institute) 1640 (Sigma-Aldrich, St. Louis, MO, USA), buffered with MOPS (morpholine propanesulphonic acid) (Sigma-Aldrich, USA), pH adjusted to 7.0 with 0.1 M NaOH, herein referred to as RPMI, was used for antifungal susceptibility testing of the yeasts by the broth microdilution method. In addition, Sabouraud dextrose agar (SDA; Difco, Becton, Dickinson and Company, Sparks, MD, USA) was used to subculture the yeast cells for inoculum preparation.

### 2.2. Antimicrobial Agents

Antifungals representing three different classes, azoles (fluconazole), polyenes (AmB), and echinocandins (caspofungin), were used in this study. Stock solutions of fluconazole (5120 µg/mL) and caspofungin diacetate (1280 µg/mL) (Sigma-Aldrich, USA) were prepared in MilliQ and then filter-sterilised. Dimethyl sulphoxide (DMSO) was used to dissolve AmB (Sigma-Aldrich, USA) to make the stock solution of 1600 µg/mL.

To test for synergy with antifungals, colistin sulphate, and two different peptidomimetics, TM8 and RK758 were included. TM8, a peptoid purchased from GaloreTx (Bangalore, Karnataka, India), was dissolved in 1× phosphate-buffered saline (PBS; pH 7.2) to make stock solutions of 2 mg/mL [31]. Another peptidomimetic, RK758, was synthesised according to the patents WO2018081869A1 and Australian Provisional Patent Application No. 2021902457) [25,40] and was made to a stock solution of 20 mM (15.2 mg/mL) in DMSO. The molecular weight and chemical structure of the antifungals are shown in Table 2. All antimicrobials were diluted in RPMI to obtain the required working concentration for the experiments.

### 2.3. Inoculum Preparation

The yeast cells, stored at −80 °C, were subcultured onto Sabouraud’s dextrose agar (SDA) (Difco, MD, USA) and incubated at 37 °C for 24 h. Three to five well-isolated colonies were then suspended in 5 mL of sterile water, and their density was adjusted to a 0.5 McFarland standard, corresponding to approximately 1–5 × 10^6^ yeast cells per mL. A 100 µL aliquot of this suspension was then added to 9.9 mL of RPMI medium, followed by further dilution in RPMI to obtain a final concentration of 5 × 10^3^ cells per mL [45] unless another concentration is mentioned.

### 2.4. Microdilution Assay for Antifungal Susceptibility Testing

The minimum inhibitory concentrations (MICs) of the antimicrobials were determined according to the Clinical and Laboratory Standards Institute (CLSI) microbroth dilution M27 standard [45] in a 96-well microtiter plate (Costar, Corning, NY, USA). After 24 h of incubation at 37 °C and 120 rpm, the optical density was measured spectrophotometrically at 530 nm. The MIC value of each drug was defined as the minimum concentration of the drug that inhibited 90% of the growth of tested yeasts. According to the CLSI M57S guidelines, epidemiological cut-off values (ECVs) for AmB were developed [46]. 

### 2.5. Checkerboard Assay to Test for Synergy

The antimicrobial synergy assay was performed using the checkerboard microdilution method in a 96-well plate, as previously described [33]. Briefly, a serial 2-fold dilution of the conventional antifungal was prepared in one plate in one direction (Plate A) and then transferred to another plate (Plate B), in which a serial dilution of the antimicrobial peptidomimetic had already been prepared in a perpendicular orientation, such that the final volume of combined agents was 100 µL per well. Subsequently, 10 µL of yeast cells maintained at 5 × 10^4^ cells per mL in RPMI, was added to each well except the blank the antimicrobial combination to make the final yeast concentration of 5 × 10^3^ cells per mL. A growth control well with no antimicrobial combination was also included. The plate was then incubated at 37 °C with shaking at 120 rpm, for 24 to 48 h. Optical density was measured using a spectrophotometer at 530 nm to determine the fractional inhibitory concentrations (FICs) of the antimicrobial combinations. Finally, the FIC index (FICI) was used to evaluate the interactions between antifungals, colistin, and peptidomimetics, categorising them as synergistic, partial synergistic, additive, indifferent, or antagonistic (Table 3). The FICI was calculated based on the MICs of individual drugs and their combinations as shown below [47]:

FICI = MIC_AB_/MIC_A_ + MIC_BA_/MIC_B_

where the following definitions hold:

MIC_AB_ is the MIC of drug A in combination with drug B;

MIC_A_ is the MIC of drug A alone;

MIC_B_ is the MIC of drug B alone;

MIC_BA_ is the MIC of drug B in combination with drug A.

Here, drug A represents an antimicrobial peptidomimetic or colistin, and drug B represents a conventional antifungal (fluconazole, AmB, or caspofungin).

**Table 3:** Interpretation of FICI [48,49,50].

FICI Range	Effect
FICI ≤ 0.5	Synergy
FICI > 0.5 to ≤0.75	Partial Synergy
FICI > 0.75 to ≤1	Additive
FICI >1 to ≤4	Indifference
FICI > 4	Antagonism

### 2.6. Time-Kill Assay 

The time-kill assay is used to measure the rate and extent of microbial killing over time with individual drugs and their combinations. It provides information on how quickly and effectively a substance can kill or inhibit the growth of a microbe. Synergy is indicated when there is a greater reduction in microbial count with the combination treatment compared to either drug alone. Since the time-kill assay requires higher cell density, *C. albicans* 002, which showed synergy with a concentration of 1–5 × 10^3^ cells per mL in the checkerboard assay, was tested at a yeast concentration of 1–5 × 10^5^ cells per mL. This was done to evaluate the combination of TM8 or RK758 with fluconazole. The FIC values obtained from these tests were used to design time-kill assays at a yeast density of 1–5 × 10^5^ cells per mL. For the time-kill assay, four Eppendorf tubes containing 5 × 10^5^ CFU/mL of *C. albicans* 002 were prepared with fluconazole, a peptidomimetic (TM8 or RK758), and their combination showing synergistic interaction in the checkerboard assay, along with a growth control. Then, 100 µL of these yeast cell suspensions, incubated at 37 °C, were plated on SDA with 0.5% Tween 80 (Chem-supply, Gillman, SA, Australia) media at predetermined time points (0 h, 3 h, 6 h, 9 h, 12 h, and 24 h) [51]. A graph was plotted to determine the change in the viable count of *Candida* over time. Synergy can be determined by comparing the difference in the number of yeast cells over time in these samples (antifungal only, peptidomimetic only, and the combination). The time-kill assay results were expressed as means ± standard errors of mean (SEMs).

### 2.7. Ergosterol Binding and Sorbitol Protection Assays

To determine the binding and sequestering efficacy of the compounds on fungal membrane ergosterol, an ergosterol binding assay was performed as previously described [52]. In brief, the MICs of TM8 and RK758 were evaluated against different yeast cells in the presence and absence of exogenous ergosterol (Sigma-Aldrich, USA) at final concentrations of 200 µg/mL and 400 µg/mL using the standard broth microdilution method outlined earlier. AmB was used as the positive control. These assays were performed in three biological replicates, each with two technical duplicates.

For the sorbitol protection assay, the MIC values for the yeasts were determined using the standard broth microdilution method, inoculating the same number of yeast cells as previously described for MIC determination. The assay was performed in three biological replicates, each with two technical duplicates, in the presence and absence of sorbitol (Sigma, USA) at a final concentration of 0.8 mol/L by incubating the microwell plates at 37 °C for seven days [53]. Caspofungin was used as the positive control. Each assay was conducted in triplicate, with two technical duplicates.

### 2.8. Cellular Leakage Effect

The effect of the peptidomimetics and AmB on the membrane of yeast was evaluated on the *C. albicans* 002 strain using a method described previously with some modifications [54]. Briefly, leakage was evaluated by measuring the absorbance of the released cellular contents upon treatment with TM8 and RK758 along with AmB, spectrophotometrically at 260 nm and 280 nm for nucleic acids and proteins, respectively. *C. albicans* 002 and *C. auris* 04 were cultured overnight in RPMI and prepared to a concentration of 10^6^ CFU/mL in PBS. One-millilitre aliquots of this suspension were treated with 1× MIC and 4× MIC of peptidomimetics (as tests) or AmB (as a positive control). After 24 h incubation at 37 °C, the samples were centrifuged at 10,621 rcf (relative centrifugal force) for 10 min. The OD of the samples was measured spectrophotometrically by pipetting 200 µL of the supernatant in each well of a UV-star plate (Greiner Bio-one GmbH, Frickenhausen, Germany). Untreated cells incubated with PBS served as the negative control. The assay was performed in three independent biological replicates with two technical duplicates.

### 2.9. Membrane-Perturbing Assay

SYTOX Green was used to assess the membrane-perturbing ability of the peptidomimetics, as previously described [55] with modifications. Briefly, *C. albicans* 002 (approximately 10^6^ CFU/mL) was suspended in PBS containing 1 µM SYTOX Green. A 100 µL aliquot was added to each well of a 96-well black plate (Nunclon Delta Surface, Thermo Fisher Scientific, Roskilde, Denmark) and incubated in the dark for 5 min. The peptidomimetics TM8 and RK758 were then added at 0.5×, 1×, and 2× MIC. Fluorescence intensity changes, indicating SYTOX Green binding to intracellular DNA, were measured every 5 min for 30 min (λ_exc_ = 485 nm, λ_ems_ = 520 nm) at 37 °C in a FLUOstar Omega microplate reader (BMG LABTECH, Ortenberg, Germany). The fluorescence of SYTOX Green was also measured for AmB treatment. Antimicrobial (peptidomimetics or AmB)-untreated cells served as negative controls. These assays were performed in three biological replicates, each with two technical duplicates.

### 2.10. Assessment of Mitochondrial Permeability Using Rhodamine 123

Rhodamine (Rh) 123 (Sigma-Aldrich, USA), a mitochondrial-specific fluorescent dye, was used to assess the impact of peptidomimetics on mitochondrial function and permeability in *C. albicans* 002 with some modifications to a previously described protocol [56]. Briefly, *C. albicans* cells in the logarithmic phase were adjusted at a concentration of approximately 10^6^ cells/mL (absorbance at 530 nm adjusted at 0.1 in PBS) and were incubated with Rh 123 (5 µM) for 15 min at 37 °C in a shaking incubator at 120 rpm. After incubation, the cells were washed 3 times with PBS to remove the extracellular Rh 123. Following this, the cells were treated with TM8 and RK758 at their 4× MIC for 15 min. A positive control was prepared with no treatment, while the negative control involved treating cells with 5 mM sodium azide. The distribution of Rh 123 in the yeast cells was examined using a Nikon AX/AS R confocal microscope (Nikon Corporation, Shinagawa-ku, Tokyo, Japan). 

### 2.11. Activity on Germ Tube Formation in C. albicans

In the presence of serum, *C. albicans* forms germ tubes, which are extensions of its cell wall that develop into tubular structures of mycelia with lengths at least equal to and breadths not exceeding half the diameter of the parent cells, with no constriction at their bases [57]. For the germ tube formation assay, *C. albicans* 002 cells were grown overnight on an SDA plate at 37 °C and then suspended in PBS and adjusted to approximately 10^6^ CFU/mL. A 50 µL aliquot of this suspension was added separately to pooled human sera and pooled mice sera at a ratio of 1:10 and incubated at 37 °C for 3 h under shaking, along with TM8 and RK758 in RPMI at 1× MIC and 2× MIC. Yeast suspensions were examined for germ tube formation. As a control, another set of sera was incubated with the same number of *C. albicans* cells, but with 50 µL of RPMI instead of peptidomimetics. The effect of peptidomimetics on *C. albicans* was evaluated by comparing the percentage of germ-tube-forming cells relative to the total cells in the presence and absence of peptidomimetics by observing the cells in brightfield illumination at a total magnification of 400× using a Leitz Orthoplan microscope (Ernst Leitz GmbH, Wetzlar, Germany). The test was performed in three technical repeats.

### 2.12. Statistical Analysis

For the cellular leakage assay, germ tube formation assay, and mitochondrial permeability using Rhodamine 123, one-way ANOVA was performed, followed by Dunnett’s post hoc test to compare each treatment group with the control group while controlling for Type I error. Two-way ANOVA was used to compare the effect of treatments on membrane permeability over time in the SYTOX Green membrane perturbing assay, followed by Dunnett’s multiple comparison test. Statistical significance was set at *p* < 0.05 (* *p* < 0.05, ** *p* < 0.01, *** *p* < 0.001, **** *p* < 0.0001). Data analysis was performed using GraphPad Prism (version 10.0.3, GraphPad Software, San Diego, CA, USA).

## 3. Results

### 3.1. MIC of Antimicrobials

The antimicrobial activities of the conventional antifungals (fluconazole, AmB, and caspofungin), peptidomimetics (TM8 and RK758), and colistin sulphate against different yeast cells are presented in Table 4. The MIC values of fluconazole, AmB, and caspofungin alone ranged from 1 to 256 µg/mL, 0.125 to 4 µg/mL, and 1 to 8 µg/mL, respectively. The MIC values of TM8 and RK758 varied from 7.8 to 31.2 µg/mL and 12 to 48 µg/mL, respectively. The MIC values of colistin ranged from 250 to 4000 µg/mL.

Following CLSI guidelines [58], resistance to fluconazole was observed in *C. albicans* 003 (MIC = 256 µg/mL) and *C. tropicalis* 001 (MIC = 8 µg/mL). In contrast, *C. albicans* 002 and *C. parapsilosis* 001 were susceptible to fluconazole. *P. kudriavzevii* is intrinsically resistant to fluconazole, and when tested, the MIC of fluconazole against *P. kudriavzevii* 001 was found to be 128 µg/mL. Neither CLSI nor the US Food and Drug Administration (FDA) have established breakpoints for AmB against *Candida* species. However, CLSI has provided ECVs, based on which *C. albicans* 002 and *C. albicans* 003 were classified as non-wild-type. In contrast, *P. kudriavzevii* 001, *C. tropicalis* 001, *N. glabratus* 001, and *C. parapsilosis* 001 were categorised as wild type strains. As there are no cut-off values for AmB against *M. guilliermondii* and *K. marxianus* by CLSI, applying the CLSI epidemiological cut-off criteria for *C. albicans* suggests that these isolates can be included as wild-type isolates.

All yeast isolates exhibited resistance to caspofungin based on CLSI breakpoints. However, CLSI does not provide interpretative criteria for *K. marxianus*. If the CLSI (2022) breakpoint for *C. albicans* is applied to *K. marxianus*, it can be considered a wild-type strain. For *C. auris*, CDC guidelines [59] were used to interpret the susceptibility, which classified *C. auris* 04 as resistant to fluconazole and AmB. 

### 3.2. Checkerboard Assay

The results of the checkerboard assay exploring the synergistic effects of antifungals (fluconazole, AmB and caspofungin) in combination with antimicrobial peptidomimetics (RK758 and TM8) and colistin against different *Candida* spp. are presented in Table 5. Overall, the combination of TM8, RK758, and colistin with conventional antifungals resulted in synergy, partial synergy, additive, and indifference effects. No antagonism was detected in any combination. 

**Figure 1:**
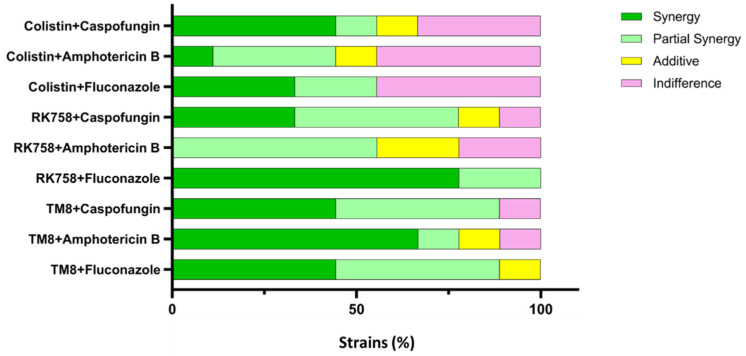
Antimicrobial combination results of AMPMs and colistin with antifungals.

Out of the nine strains tested, TM8 showed synergy with both fluconazole and caspofungin, with 44.4% synergy and 44.4% partial synergy for each. However, RK758 enhanced the activity of fluconazole in six cases, with two strains showing partial synergy, suggesting that RK758 is more effective in synergising with fluconazole than TM8. On the other hand, TM8 demonstrated superior synergy with AmB, achieving 66.7% synergy and 11.1% partial synergy, whereas RK758 exhibited only partial synergy with AmB in 66.7% of isolates, with the remaining cases showing additive (22.2%) or indifference (22.2%) effects. Regarding caspofungin, RK758 showed synergy in 33.3% of cases and partial synergy in 44.4%. Meanwhile, colistin sulphate displayed indifferent activity in 44.4% against both fluconazole and AmB but showed a synergistic combination with caspofungin in 44.4% of cases, while 33.3% showed indifference (Figure 1).

### 3.3. Time-Kill Assay

The MICs for the antimicrobials at 10^5^ CFU/mL for *C. albicans* 002 were as follows: fluconazole (4 µg/mL), TM8 (32.5 µg/mL), and RK758 (48 µg/mL). When a checkerboard assay was performed for this concentration of yeast cells, TM8 and fluconazole showed synergy at 7.8 µg/mL and 1 µg/mL, respectively. Similarly, it was 12 µg/mL and 1 µg/mL for RK758 and fluconazole. 

**Figure 2:**
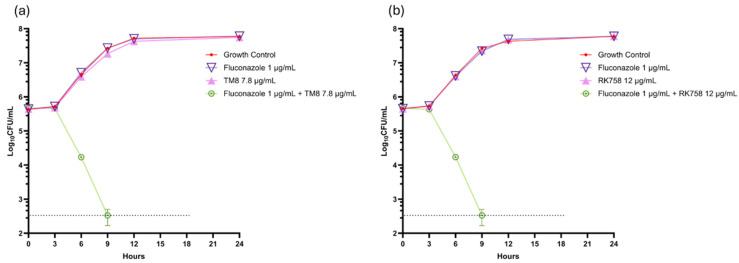
Time-kill curve plots of fluconazole, antimicrobial peptidomimetics (TM8 or RK758), and their combinations against *C. albicans* 002 (**a**) with TM8 (**b**) with RK758. The black dotted line represents the limit of detection. The data are expressed as mean ± SEM of triplicates.

When a time-kill curve was plotted for *C. albicans* 002 at the synergistic concentration of fluconazole and TM8, the combination displayed fungicidal activity, achieving a 3 log_10_ reduction in the CFU/mL over 24 h, with samples taken every three hours for a viable count by plating on a medium, as shown in Figure 2a. In contrast, when fluconazole and TM8 were tested individually at their sub-MICs, they were ineffective, with viable counts increasing from 10^5^ to nearly 10^8^ CFU/mL. This indicates that neither antimicrobial at a sub-MIC alone inhibited the growth of *C. albicans*; however, their combination demonstrated continuous inhibition of colony counts compared to either antimicrobial individually or the growth control (*p* < 0.05), highlighting their synergistic activity. Similar results were observed when RK758 and fluconazole were used in combination at sub-MICs, as shown in Figure 2b. Neither antimicrobial alone at the sub-MIC could prevent the growth, but their combination showed inhibition of the yeast cell, further supporting the synergy between these antimicrobials.

### 3.4. Ergosterol Binding and Sorbitol Protection Assays

For all yeast cells, the MIC of both TM8 and RK758 increased by 4- to 8-fold in the presence of either concentration of ergosterol, suggesting that both peptidomimetics act on the fungal membrane (Table 6). As a control, AmB was tested for its MIC against *C. albicans* 002 and *C. auris* 04 at both concentrations of ergosterol, and the MIC of the peptidomimetics was determined against these strains. An eight-fold increase in MIC was observed for AmB against *C. albicans* 002 and *C. auris* 04 at both the 200 µg/mL and 400 µg/mL ergosterol concentrations. The MIC of caspofungin remained unchanged in the presence of sorbitol (0.8 M) for all strains on both the second and the seventh days of incubation, as shown in Supplementary Table S1.

### 3.5. Cellular Leakage Assay

**Figure 3:**
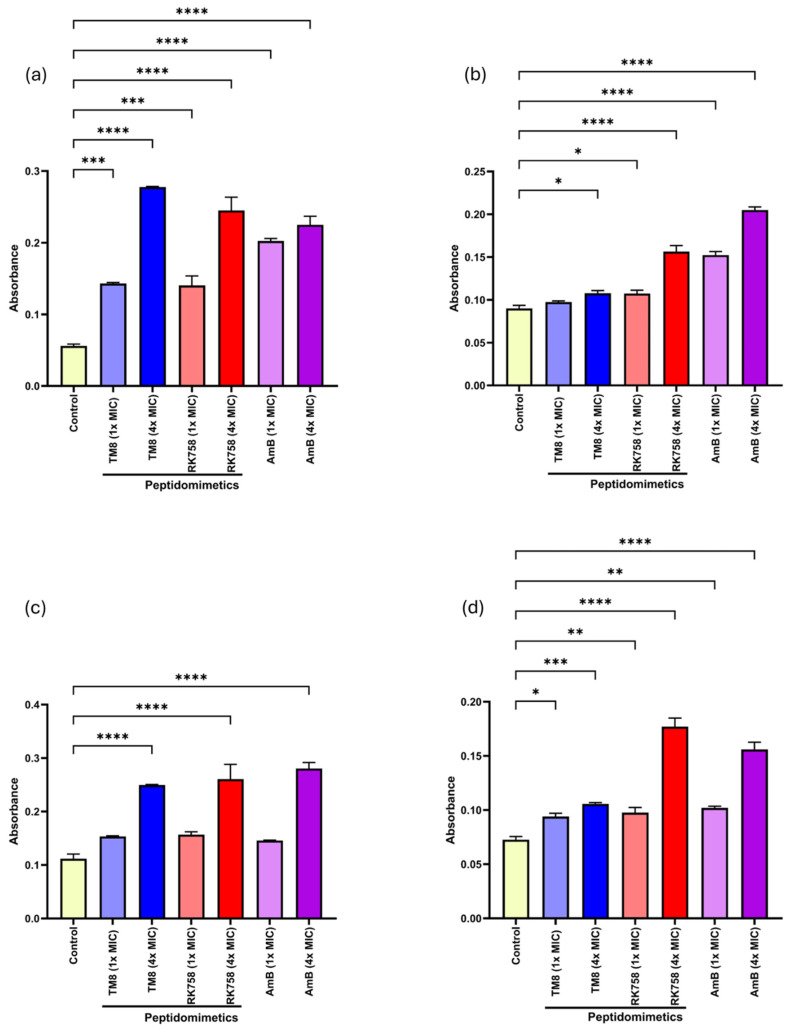
Cellular leakage of *C. albicans* 002 measured at (**a**) 260 nm and (**b**) 280 nm and *C. auris* 04 measured at (**c**) 260 nm and (**d**) 280 nm, corresponding to nucleic acids and proteins, respectively. Ordinary one-way ANOVA was performed, followed by Dunnett’s multiple comparison test to assess statistical significance. * *p* < 0.05, ** *p* < 0.01, *** *p* < 0.001, **** *p* < 0.0001.

The intracellular components of yeast cells that absorb at 260 nm (nucleotides) and 280 nm (proteins) in the cell supernatant were measured spectrophotometrically for *C. albicans* 002 and *C. auris* 04 (Figure 3a–d). A significant release of nucleic acids was observed at 4× MIC for both peptidomimetics in both strains. At 1× MIC of all the antimicrobials, there was no appreciable release of nucleic acid in the case of *C. auris* 04. In contrast, intracellular protein content was released at both concentrations of antimicrobials in *C. auris* 04. For both peptidomimetics, a concentration-dependent release of intracellular components was observed. Similar results were obtained with the positive control, AmB.

### 3.6. Membrane-Perturbating Assay

**Figure 4:**
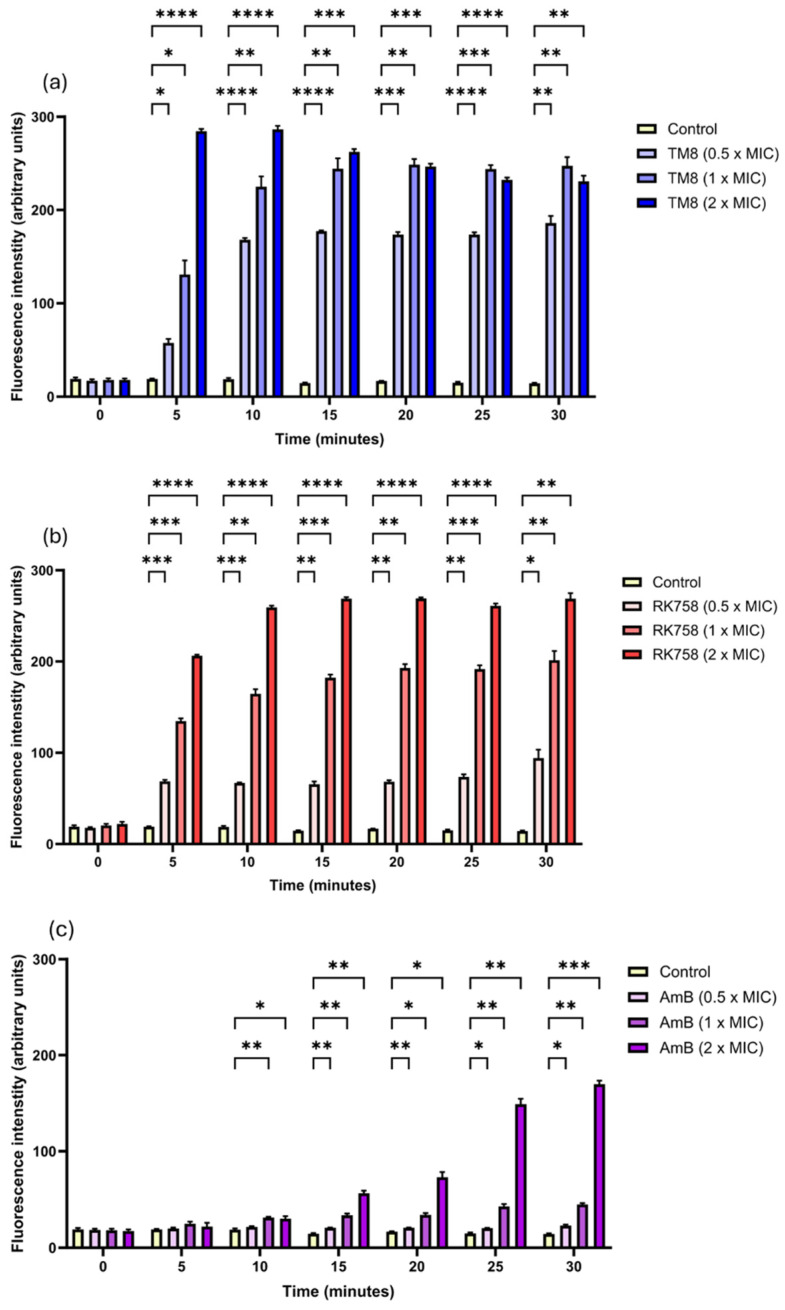
Analysis of membrane permeabilization caused by antimicrobials assessed by SYTOX Green fluorescence. Treatment by (**a**) TM8, (**b**) RK758, and (**c**) AmB. Two-way ANOVA with Geisser–Greenhouse correction was performed to assess how different treatments affect membrane permeability over time, followed by Dunnett’s multiple comparison test to compare each treatment to the control group. Statistical significance is indicated in the graph only for those conditions that showed significant differences. * *p* < 0.05, ** *p* < 0.01, *** *p* < 0.001, **** *p* < 0.0001.

The integrity of the yeast cell membrane was assessed following treatment with the peptidomimetics using the fluorescent dye SYTOX Green. The dye normally does not penetrate intact cells. It forms complexes with DNA only when the membrane is compromised or disrupted. When the dye forms a complex with DNA, its fluorescence increases compared to that in the aqueous solution or when the yeast cells are not treated with the membrane-permeabilising agent, or when the treating agent does not permeabilise the cell. The time course of SYTOX Green uptake during a 30 min period on exposure to TM8, RK758, and AmB at 1× MIC and 2× MIC was monitored by measuring the fluorescence every 5 min (Figure 4a–c). There was a time- and concentration-dependent increase in membrane perturbation in *C. albicans* 002 strain for both RK758 and AmB, while the time-dependent increase in SYTOX Green fluorescence was less pronounced with TM8-treated cells at 1× MIC (Figure 4a–c). 

### 3.7. Mitochondrial Permeability Using Rhodamine 123

**Figure 5:**
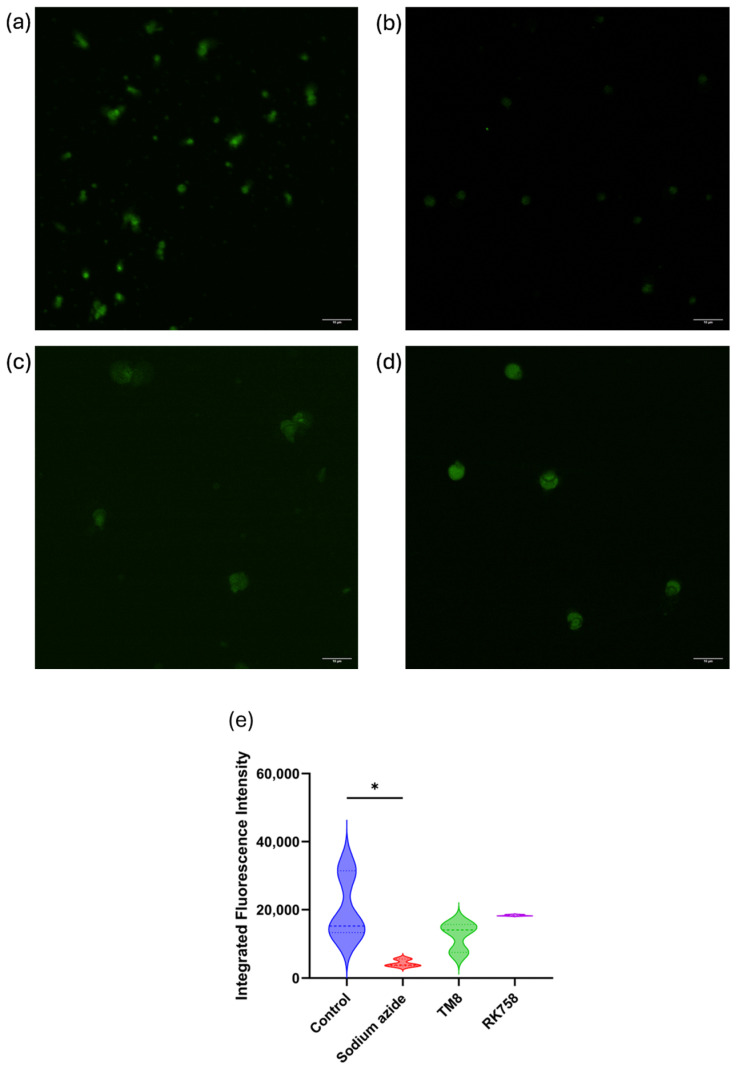
*C. albicans* 002 mitochondrial staining to measure the membrane potential by Rhodamine 123 in (**a**) control, (**b**) sodium azide-treated, (**c**) TM8-treated, and (**d**) RK758-treated cells and (**e**) violin plot showing the data distribution and statistical significance. The scale bar in all panels of (**a**–**d**) represents 10 μm. * *p* < 0.05.

Images of Rh 123-stained *C. albicans* 002 are presented, depicting untreated control cells (Figure 5a), sodium azide-treated cells (Figure 5b), TM8-treated cells (Figure 5c), and RK758-treated cells (Figure 5d) to assess the mitochondrial permeability. The integrated fluorescence intensity was quantified (Fiji/ImageJ) for untreated as well as treated cells with the presentation of data distribution using a violin plot (Figure 5e). In the Shapiro–Wilk test, the data were confirmed to have a normal distribution (*p* > 0.05). Therefore, an ordinary one-way ANOVA, followed by Dunnett’s post hoc test, was performed for multiple comparisons. Statistical analysis revealed a significant reduction in fluorescence in sodium azide-treated cells, while no statistical difference was observed in TM8 (*p* = 0.2815)- or RK758 (*p* = 0.9638)-treated yeast cells compared to the untreated control (*p* = 0.0199).

### 3.8. Germ Tube Formation Assay

**Figure 6:**
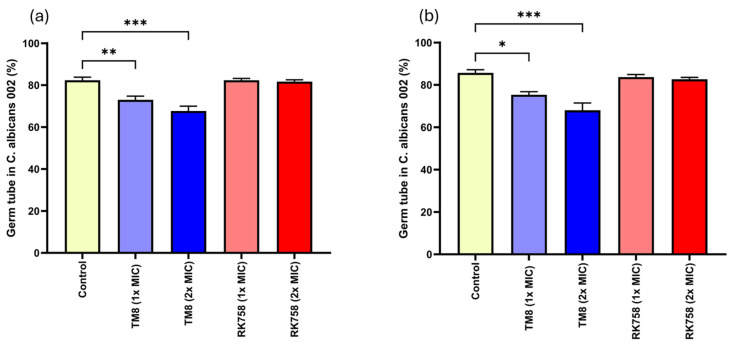
Germ tube formation in *C. albicans* 002 on treatment with the peptidomimetics in pooled sera of (**a**) mice and (**b**) humans. All values are expressed as mean ± SEM (n = 3). Statistical significance was determined by one-way ANOVA followed by Dunnett’s multiple comparison test. *p* values are shown only for the statistically significant conditions. * *p* < 0.05, ** *p* < 0.01, *** *p* < 0.001.

Germ tube formation was assessed in the presence and absence of peptidomimetics. RK758, at both 1× and 2× MIC, did not inhibit germ tube formation in *C. albicans* in either mouse or human sera. The percentage of germ tube formation ranged from 82% to 84%, which was comparable to the untreated serum samples (82% in mouse sera and 86% in human sera). On the other hand, TM8 reduced germ tube formation to 73% and 68% in mice sera and 75% and 68% in human sera at 1× MIC and 2× MIC, respectively (Figure 6).

## 4. Discussion

The increasing prevalence of systemic manifestations of candidiasis is driven by high-risk conditions including cancer, haematological pathology, indwelling medical devices, immunosuppressant use, and broad-spectrum antibiotic therapy. Further, the increasing trend of resistance to conventional antifungals emphasises the urgent need for effective alternative therapeutic strategies. One promising approach is combination therapy, which has demonstrated effectiveness in improving treatment outcomes and overcoming resistance [60]. In this study, the MIC of selected antimicrobials was first evaluated against different yeast cells, followed by a synergy test to evaluate the efficacy of combining conventional antifungals with antimicrobial peptidomimetics.

Fluconazole is one of the most prescribed antifungals for *Candida* infections. It disrupts the ergosterol biosynthetic pathway by specifically binding to and inhibiting lanosterol 14α-demethylase (Erg11), an enzyme responsible for the oxidative removal of the 14α-methyl group from lanosterol. This disruption compromises cell membrane integrity and inhibits cell growth [61]. However, as yeasts are not killed, prolonged infections can lead to resistance, reducing the efficacy of fluconazole in severe *Candida* infections. Consequently, there has been an increasing prevalence of fluconazole-resistance in *Candida* spp. [62]. Another yeast, *N. glabratus*, demonstrates reduced susceptibility or inherent resistance to fluconazole, and if it exhibits a susceptible-dose-dependent response towards it, a high dose (800 mg/day) is recommended [63]. It further exhibits cross-resistance to other azoles [64]. Another medically important yeast, *P. kudriavzevii*, is intrinsically resistant to fluconazole due to a mutation in the lanosterol 14α-demethylase, which reduces its susceptibility to azoles. Additionally, the overexpression of the ABC2 efflux pump contributes to this resistance [65]. In the current study, fluconazole had an MIC_50_ = 8 μg/mL and MIC_90_ = 128 μg/mL considering different yeast cells, implying insufficiency as monotherapy. These findings show increasing ineffectiveness of fluconazole among medically important yeasts. 

Echinocandins, such as caspofungin, are frontline antifungal treatment for candidaemia due to their fungicidal nature, low toxicity compared to other conventional antifungals, and broad-spectrum activity against invasive *Candida* spp. such as *C. albicans*, *C. tropicalis*, *P. kudriavzevii*, and *C. auris* [66]. These antifungal drugs inhibit (1,3)-β-glucan synthesis, which is involved in the biosynthesis of the *Candida* cell wall [21]. In the current study, most isolates were resistant to caspofungin, with MICs ranging from 1 to 8 μg/mL and a geometric mean MIC of 2.38 μg/mL. Resistance to caspofungin is due to point mutations in the hotspot 1 (HS1) and hotspot 2 (HS2) regions of the *FKS1* and *FKS2* genes. These mutations lead to an altered conformation of the β-1,3-D-glucan synthase (FKS) enzyme, which is the primary target of caspofungin, thereby reducing its capacity to inhibit the biosynthesis of β-1,3-D-glucan, an essential component of the cell wall [65]. Some species, such as *C. parapsilosis* and *M. guilliermondii*, have been documented to be intrinsically less susceptible to echinocandins [67], and there is a growing emergence of higher-order resistance in *N. glabratus* [68] and *C. albicans* [69], with reports of co-resistance to fluconazole [63]. Similarly, a study on *C. auris* candidaemia conducted in India reported a high MIC_90_ for caspofungin (8.0 μg/mL) [70]. 

In the current study, the MIC_90_ of AmB against yeast cells was found to be 4 μg/mL. However, the efficacy of AmB against *C. albicans* and *C. auris* was less promising. Amphotericin B is a fungicidal, amphipathic, polyene drug that targets ergosterol in the fungal cell membrane [71]. By binding to ergosterol, it forms pores in the membrane, compromising cell integrity and ultimately causing cell death [71]. However, different yeasts, such as *C. lusitaniae* and *M. guilliermondii*, exhibit a rapid and innate acquisition of AmB resistance. Similarly, *N. glabratus* and *P. kudriavzevii* have been found to be less susceptible in several studies [16,72]. In *P. kudriavzevii*, although resistance to AmB is rare, it can occur due to the inactivation of the *ERG3* gene resulting in decreased production of ergosterol, which is replaced by 14α-methylfecosterol in the cell membrane, thereby making the drug ineffective [65]. 

Given the increasing rates of antifungal resistance and reduced drug sensitivity, these different classes of antifungals as a monotherapy are questioned, as a higher dose is required for therapeutic success. These reports warrant the necessity for potent antifungal alternatives. The peptidomimetics consistently demonstrated antifungal activity against all yeasts, regardless of their susceptibility to conventional antifungals or whether they were wild-type or non-wild-type strains. The antifungal activity of peptidomimetics against isolates resistant to fluconazole and/or caspofungin suggested one or more modes of action that could be different from those of antifungals. Similarly, TM8 and RK758 had MICs from 15.6 to 31.2 µg/mL for yeast pathogens in which AmB was not relatively active (MIC = 4 µg/mL). TM8 showed a higher MIC only against *C. auris* 04 (31.2 µg/mL), while RK758 exhibited a higher MIC against *C. parapsilosis* 001 (62.5 µg/mL). However, for all other isolates, both peptidomimetics exhibited consistently lower MICs, indicating their potential as promising antifungal agents (7.8–15.6 µg/mL). 

Antimicrobial peptides and their peptidomimetics not only exhibit antifungal activity but can also synergise with conventional antifungal drugs [33]. Considering new antifungal strategies, combination therapy is preferred due to its ability to reduce the risk of antifungal resistance, enhance efficacy at sub-MICs of individual drugs, shorten treatment duration, and lower drug toxicity [73]. Studies have reported a 90–100% synergistic effect, with MIC reductions of 4- to 8-fold compared to individual antifungal drugs or AMPs alone [74]. The current study primarily investigated the antimicrobial peptidomimetics TM8 and RK758 to assess possible mechanisms of action against yeasts and their synergy with conventional antifungals. 

In the current study, the MIC of TM8 and RK758 increased by at least 4-fold in the presence of exogenous ergosterol. Ergosterol is the major sterol of the fungal cell membrane. The ability of any compound’s membrane destabilization capability can be assessed by showing that it can bind to exogenous ergosterol added to the cell suspension. Exogenous ergosterol in the medium competitively binds to the antimicrobial capable of binding cell-membrane-bound ergosterol. Consequently, the antifungal activity of the test agent reduces with the rise in MIC [75]. In contrast, no change in the MIC was seen in the presence of sorbitol. Sorbitol acts as an osmotic protectant, stabilizing fungal protoplasts. In its presence, the MIC of a compound targeting the fungal cell wall increases to a much higher value. The protective effect of sorbitol as an osmoprotectant is not exclusive to β-(1,3) glucan synthesis inhibitors; it extends to inhibitors of other cell wall polymers, and mechanisms associated with cell wall synthesis too [53]. This implies that both the compounds can act by binding with the ergosterol in the cell membrane of yeast cells, conferring a similar mechanism to AmB, and probably do not act on the cell wall, unlike echinocandins. The membranolytic effects of the peptidomimetics were further examined by performing an intracellular component leakage assay and found that after TM8 and RK758 treatment, proteins and nucleic acids are released from the cells, based on their absorbance at 260 nm and 280 nm, as a marker of cell lysis. This is further supported by a positive SYTOX Green assay, which is based on the principle that this fluorescent dye cannot bind with DNA unless the membrane is disrupted. The membrane-specific activity of the peptidomimetics can be further studied and complemented by a bis(1,3-dibutylbarbituric acid) trimethine oxonol (DiBAC_4_(3)) assay, live–dead staining with SYTO 9 and propidium iodide, and electron microscopy [33,76].

In the mitochondrial permeability assay using Rhodamine 123, there was no statistically significant difference in the fluorescence intensity between untreated and peptidomimetic-treated *C. albicans* 002 suggesting that there is no mitochondrial respiration suppression during the short treatment period of 15 min at 4× MIC. The untreated control showed a strong fluorescence intensity of Rhodamine 123, suggestive of intact membrane potential. In contrast, sodium azide-treated cells showed a marked reduction in fluorescence, consistent with mitochondrial membrane depolarization. Though there was a decrease in fluorescence in TM8-treated yeast cells, it was not statistically significant. Further time points should be examined for mitochondrial permeability after exposing the yeast cells for a longer period. 

*C. albicans* is a polymorphic fungus capable of exhibiting different morphological forms, including spherical yeast, elongated pseudohyphae, filamentous hyphae, and chlamydospores, and shows phenotypic switching. The yeast form is primarily associated with dissemination, whereas the filamentous form facilitates tissue invasion. The ability of *C. albicans* to form a cellular extension in the form of germ tubes is an important virulence factor in its pathogenesis that helps in its resistance to phagocytosis and invasion [77]. In this study, TM8 was found to inhibit germ tube formation in *C. albicans* in a dose-dependent manner, reducing it by 9 to 14% in human sera and 11% to 18% in mice sera. In contrast, RK758 had no such effect. This suggests that TM8 may also function as an anti-virulence agent against *C. albicans.* Conversely, RK758 lacks this activity, or its efficacy in inhibiting germ tube formation is reduced due to potential binding with serum proteins. However, antimicrobial peptides like CGA-N12 (NH_2_–ALQGAKERAHQQ–COOH) have been shown to be more effective, inhibiting the germ tube formation by more than 50% [78]. Additionally, the frog skin peptide temporin G inhibited germ tube formation in a dose-dependent manner, with 31%, inhibition at 1× MIC, 72% at 2×, and 90% at 4× MIC [79].

In the current study, when there was a synergistic interaction, the activity of fluconazole was significantly enhanced when combined with the TM8 or RK758, leading to a 4- to 128- or 256-fold reduction in the fluconazole MIC. Even in *P. kurdiavzevii*, which is inherently resistant to fluconazole, TM8 and RK758 reduced the MIC of fluconazole by 64 and 8 times. In synergistic interactions, when peptidomimetics were used in concentrations below their MICs, they could inhibit the growth of yeast cells. Overall, in nine yeasts, with respect to any of the three antifungals, TM8 and RK758 exhibited synergy in 51.9% (14/27) and 33.3% (9/27) combinations, respectively, whereas partial synergy was seen in 33.3% (9/27) and 40.7% (11/27), respectively. Such synergy was seen in both antifungal-sensitive and -resistant yeast isolates. In *C. albicans* 002, the synergistic activity due to TM8 on caspofungin was sufficient to overcome caspofungin resistance by reducing the MIC below the breakpoint value, thus providing potential therapeutic efficacy. Similar results were obtained for fluconazole, AmB, and caspofungin in the case of *C. albicans* 003 due to TM8. It further showed such combinational benefit for *C. auris* 04 by bringing down the MIC value of both fluconazole and AmB from the non-wild-type category to the wild type. Similarly, RK758 played such a synergistic role in the case of *C. albicans* 003 for fluconazole. However, there was no uniform pattern of synergy between the combinations, thus implying strain-dependent activity. While the precise mechanism of synergy is unknown, based on our findings on their ergosterol binding activity, and hence action on the cell membrane, we hypothesise these peptidomimetics enhance antifungal activity by disrupting cell membrane integrity, thereby allowing more effective penetration or improving antifungal interaction with its target site(s) in yeast cells. Furthermore, these peptidomimetics may act not only on the fungi cell membrane, but may also exert multiple underlying mechanisms contributing to their synergistic interactions. Apart from being membrane-active, AMPs or their mimetics can target the cell wall by inhibiting the synthesis of glucan (e.g., pneumocandins, aculeacins), chitin (e.g., nikkomycin Z, polyoxins, defensins, arthrichitin), and mannan (e.g., pradimicins, benonomicins). Although our peptidomimetics are less likely to act on the cell wall, as demonstrated by the sorbitol protection assay, other potential modes of action include nucleic acid inhibition (actinomycin D, indolicidin, buforins), antibiofilm activity (e.g., leucinostatin A), and germ tube inhibition (e.g., fengycins, surfactins) [80]. 

In the checkerboard assay, no antagonism was found between any of the combinations of peptidomimetics and the conventional antifungals. Conventional antifungals have been shown to act synergistically with antimicrobial peptides. For instance, the combined effect of AMPs with fluconazole against *Candida* produced synergy in 70–90% of isolates, additive effects in 10–20%, and indifference in up to 10%. Amphotericin B, when combined with AMPs (HNP-1, HNP-3, and His 5), revealed synergy in 90% of isolates and additive effects in the remaining 10%. Additionally, a 100% synergistic effect was found when AmB was combined with AMPs (hBD-1, -2, and -3 and HNP-2). Caspofungin combined with several AMPs resulted in a 100% synergistic effect [74]. However, susceptibility to proteolysis is the major limitation of AMPs for therapeutic purposes that is overcome by the antimicrobial peptidomimetics [81]. 

Polymyxin antibiotics, such as colistin sulphate, are bactericidal compounds that target Gram-negative bacteria and act by disrupting the bacterial cell membrane [4]. Colistin is ineffective against *Candida* spp. as a monotherapy. The current results indicated synergy with colistin in 30% of combinations with either of the antifungals, while partial synergy was observed in 22% of cases. Synergistic effects have also been demonstrated in previous studies of colistin used in conjunction with conventional antifungal drugs such as azoles, echinocandins, and AmB [30]. It has been proposed that the mechanism of synergy is due to the positively charged colistin binding to the fungal cell membrane and increasing membrane permeability for the antifungal drugs. These drugs then amplify the membrane disruption and produce their individual antifungal effect, ultimately leading to cell death. Previous studies researching the synergistic effect of colistin with fluconazole proposed that colistin is able to bind and disrupt the cell membrane more effectively when it is depleted of ergosterol [30]. Other studies have proposed that the synergy between caspofungin and colistin is due to caspofungin altering the cell wall, which facilitates the access of colistin to the fungal membrane [82]. Similarly, another study hypothesised that AmB acts to permeabilise the cell membrane, which aids further membrane disruption by colistin [83]. Meanwhile, several studies have demonstrated increased cytotoxicity, particularly with colistin combinations that have been associated with high nephrotoxicity risks [84]. In our study, the synergistic combination concentration of colistin with the antifungals was in the range of 7.8 to 1000 µg/mL, which was still high for use for therapeutic purposes.

A time-kill assay was also used in the current study to test the synergistic effect of fluconazole with TM8 as well as RK758. Our study demonstrated consistent synergistic outcomes in both the checkerboard and time-kill assays for a fluconazole-resistant *C. albicans* isolate (*C. albicans* 002). Such agreement between methods reinforces the potential effectiveness of the peptidomimetics and fluconazole against the isolate [85]. Individually, each drug was ineffective against the tested *Candida* spp., but at the same ineffective concentration levels, their combination exhibited fungicidal activity, preventing *C. albicans* regrowth over 24 h with a 3-log reduction in CFU/mL. Though fluconazole is fungistatic, its combination with peptidomimetics induced fungicidal effects. Similar studies have shown that synergistic combinations can convert fungistatic drugs into fungicidal ones, providing a more rational therapeutic option [86]. This demonstrates the significance of combination therapy, particularly antifungals with peptidomimetics. Pankey et al. (2014) tested the combination of polymyxin B and fluconazole and found synergy (60%) and fungicidal activity (48%) against *N. glabratus* isolates [51]. 

In the present study, although the toxicity profile of the antimicrobial combinations was not assessed, previous reports have established the 50% hemolysis dose (HD_50_) of TM8 and colistin against horse erythrocytes to be 27.2 μg/mL and >256 μg/mL [33]. Similarly, the CC_50_ (concentration causing 50% cytotoxicity) of RK758 was determined to be 111.0 μg/mL and 115.0 μg/mL against MDCK and Vero cell lines, respectively [28], while its HD_50_ was >300 μg/mL (unpublished data). In future studies, comprehensive cytotoxicity analyses of these peptidomimetics across a broader range of cell lines, both individually and in combination with conventional antifungals will be performed. These studies will help establish detailed therapeutic indices at synergistic concentrations, thereby offering insights into the safety profiles of these combinations across different cell types.

## 5. Conclusions

The emergence of yeast pathogens causing invasive MDR infections emphasises the urgent need to identify effective treatment. This research demonstrates successful combination therapies of antimicrobial peptidomimetics with conventional antifungal drugs such as fluconazole, AmB, and caspofungin against yeasts in vitro. The combinations produced synergy without antagonism, which is a prerequisite for the development of potential combination therapy; however, some combinations showed either additive effects or indifference as well. Though colistin sulphate exhibited synergy in some cases, its concentration that exhibited synergy remained too high for in vivo use. Based on the antimicrobial synergy observed in this study and the safety profiles of individual compounds from our previous studies, we recommend advancing these combination therapies for further evaluation. This includes testing across a broader range of cell lines to evaluate combination-specific cytotoxicity, as well as conducting in vivo studies to assess the compounds’ antifungal efficacy and overall safety. From a mechanistic perspective, both peptidomimetics targeted the cell membrane and exhibited concentration-dependent activity, as demonstrated by the SYTOX Green uptake and cellular leakage assays. Further investigation into the specific mechanism of action of combination therapy of peptidomimetics and conventional antifungals against yeast pathogens is required. Similarly, the cytotoxicity of the peptidomimetics combined with antifungals should also be assessed. These findings provide a foundation for future research and potential clinical applications in combating antifungal resistance, as synergistic interactions allow for the use of lower drug doses to effectively treat *Candida* and other yeast infections, reducing toxicity and adverse effects.

## 6. Limitations

The toxicity of antimicrobial combinations was not investigated in this study. Since these experiments were conducted in vitro, they may not necessarily translate to in vivo synergy outcomes. It is also possible that these peptidomimetics may have multiple mechanisms of action, which have not been extensively studied. Additionally, further detailed mechanistic investigations are recommended to understand the mechanism of synergistic outcomes of peptidomimetics and antifungals. The yeast cells were not subjected to a resistance evolution assay using the synergistic combination of antimicrobials.

## Figures and Tables

**Table 1:** Fungal isolates used in this study.

Yeasts	Lab Designation	Strain ID	Nature of Strain	Isolation Source	Country of Isolation
*Candida albicans*	*C. albicans* 002 [34,35]	GRI 681	WHO critical-priority; adherent strain	Cervix from an asymptomatic woman	UK
	*C. albicans* 003 [35,36]	Y5499	WHO critical-priority; strain showing resistance to complement attack but unable to grow in serum	Human saliva	Australia
*C. tropicalis*	*C. tropicalis* 001 [35,36,37]	N/A	WHO high-priority; strain aggregating platelets within the shortest time among other non-*albicans/auris Candida*	Human saliva	Australia
*C. parapsilosis* complex	*C. parapsilosis* 001 [35,36,37]	Y316	WHO high-priority; recently recognised as a complex of 4 species: *C. parapsilosis*, *C. orthopsilosis*, *C. metapsilosis*, *Lodderomyces elongisporus*	Human saliva	Australia
*Meyerozyma guilliermondii*	*C. guillermondii* 001 [16,36,37,38]	Y324	This species is constitutively less susceptible to polyenes and echinocandins as compared to other yeast-like fungi	Human saliva	Australia
*Nakaseomyces glabratus*	*C. glabrata* 001 [8,35]	N/A	WHO high-priority	Human saliva	Australia
*Pichia kudriavzevii*	*C. krusei* 001 [35,36,37]	Y301	WHO medium-priority; strong capacity for adhering to polystyrene and acrylic surfaces	Human saliva	Australia
*Kluyveromyces marxianus*	*C. kefyr* 001 [36,37]	Y83	High capacity for adhering to buccal mucosa among non-*albicans Candida*	Human saliva	Australia
*Candidozyma auris*	*C. auris* 04 [9,35,39]	AR0384	WHO critical-priority; aggregative strain	Blood	South Africa

N/A = not available.

**Table 2:** Antimicrobial classes, molecular weights, and structures.

Antifungal Class	Antifungal	Molecular Weight	Structure
Azoles	Fluconazole [41]	306.27 g/mol	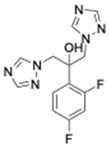
Echinocandins	Caspofungin [42]	1093.3 g/mol	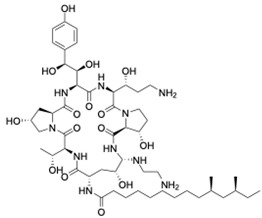
Polyenes	Amphotericin B [43]	924.1 g/mol	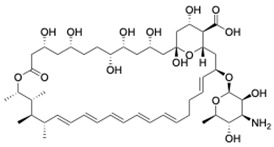
**Antibiotic Class**	**Antibiotic**	**Molecular Weight**	**Structure**
Polymyxin	Colistin [44]	1155.4 g/mol	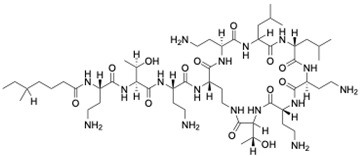
**Peptidomimetic**		**Molecular Weight**	**Structure**
RK758 [25]		759.71 g/mol	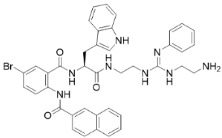
TM8 [31,33]		1115.52 g/mol	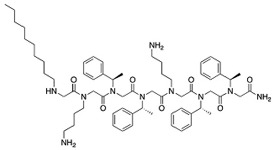

**Table 4:** MIC of conventional antifungals (fluconazole, amphotericin B, and caspofungin), antimicrobial peptidomimetics, and colistin against *Candida* spp.

*Candida* Species	MIC (µg/mL)					
	Fluconazole	AmB	Caspofungin	Colistin	TM8	RK758
*C. albicans* 002	2	4	2	2000	15.6	12
*C. albicans* 003	256	4	2	2000	15.6	24
*P. kudriavzevii* 001	128	2	2	4000	7.8	12
C. *tropicalis* 001	8	0.5	2	250	7.8	12
*N*. *glabratus* 001	8	0.25	2	2000	15.6	12
*M*. *guilliermondii* 001	4	0.125	4	2000	7.8	12
*C. parapsilosis* 001	1	0.25	8	2000	7.8	48
*K. marxianus* 001	1	0.25	2	2000	7.8	12
*C*. *auris* 04	128	4	1	1000	31.2	12
MIC_50_	8	0.5	2	2000	7.8	12
MIC_90_	128	4	8	2000	31.2	24
Geometric mean MIC of the antifungals against *Candida* spp.	13.5	0.8	2.3	1587.4	11.5	15.1

**Table 5:** Synergistic effects of antifungals in combination with peptidomimetics and colistin in checkerboard assay.

Strains	Antimicrobial Combinations	MIC (µg/mL)				FIC		FICI	Interpretation
		Antifungal	Antifungal in Combination with Peptidomimetic/Colistin	Peptidomimetic/Colistin	Peptidomimetic/Colistin in Combination with Antifungal	Antifungal	Peptidomimetic/Colistin		
*C. albicans* 002	Fluco + TM8	2	0.25	15.6	3.9	0.125	0.25	0.38	Synergy
	Fluco + RK758	2	0.125	12	4	0.0625	0.25	0.31	Synergy
	Fluco + Colistin	2	0.125	2000	62.5	0.0625	0.031	0.094	Synergy
	AmB + TM8	4	0.002	15.6	15.6	0.0005	1	1.0005	Indifference
	AmB + RK758	4	0.125	12	6	0.031	0.5	0.53	P. Synergy
	AmB + Colistin	4	4	2000	2000	1	1	2	Indifference
	Caspo + TM8	2	0.25	15.6	3.9	0.125	0.25	0.38	Synergy
	Caspo + RK758	2	0.5	12	3	0.25	0.25	0.50	Synergy
	Caspo + Colistin	2	2	2000	250	1	0.125	1.13	Indifference
*C. albicans* 003	Fluco + TM8	256	2	15.6	3.9	0.0078	0.25	0.26	Synergy
	Fluco + RK758	256	1	24	6	0.0039	0.25	0.25	Synergy
	Fluco + Colistin	256	256	2000	2000	1	1	2	Indifference
	AmB + TM8	4	1	15.6	0.98	0.25	0.063	0.31	Synergy
	AmB + RK758	4	2	24	6	0.5	0.25	0.75	P. Synergy
	AmB + Colistin	4	4	2000	2000	1	1	2	Indifference
	Caspo + TM8	2	0.25	15.6	3.9	0.125	0.25	0.38	Synergy
	Caspo + RK758	2	0.5	24	6	0.25	0.25	0.50	Synergy
	Caspo + Colistin	2	2	2000	1000	1	0.5	1.50	Indifference
*P. kudriavzevii* 001	Fluco + TM8	128	2	7.8	3.9	0.016	0.5	0.52	P. Synergy
	Fluco + RK758	128	16	12	3	0.125	0.25	0.38	Synergy
	Fluco + Colistin	128	32	4000	1000	0.25	0.25	0.50	Synergy
	AmB + TM8	2	0.25	7.8	1.95	0.125	0.25	0.38	Synergy
	AmB + RK758	2	2	12	12	1	1	2	Indifference
	AmB + Colistin	2	1	4000	1000	0.5	0.25	0.75	P. Synergy
	Caspo + TM8	2	1	7.8	1.95	0.5	0.25	0.75	P. Synergy
	Caspo + RK758	2	1	12	6	0.5	0.5	1	Additive
	Caspo + Colistin	2	2	4000	4000	1	1	2	Indifference
*C. tropicalis* 001	Fluco + TM8	8	0.5	7.8	3.9	0.063	0.5	0.56	P. Synergy
	Fluco + RK758	8	0.031	12	6	0.0039	0.5	0.504	P. Synergy
	Fluco + Colistin	8	0.015	250	62.5	0.0019	0.25	0.25	Synergy
	AmB + TM8	0.5	0.125	7.8	1.95	0.25	0.25	0.50	Synergy
	AmB + RK758	0.5	0.25	12	0.75	0.5	0.063	0.56	P. Synergy
	AmB + Colistin	0.5	0.25	250	62.5	0.5	0.25	0.75	P. Synergy
	Caspo + TM8	2	0.5	7.8	1.95	0.25	0.25	0.5	Synergy
	Caspo + RK758	2	1	12	3	0.5	0.25	0.75	P. Synergy
	Caspo + Colistin	2	0.06	250	7.8	0.03	0.031	0.061	Synergy
*N. glabratus* 001	Fluco + TM8	8	4	15.6	7.8	0.5	0.5	1	Additive
	Fluco + RK758	8	2	12	6	0.25	0.5	0.75	P. Synergy
	Fluco + Colistin	8	8	2000	2000	1	1	2	Indifference
	AmB + TM8	0.25	0.125	15.6	7.8	0.5	0.5	1	Additive
	AmB + RK758	0.25	0.125	12	6	0.5	0.5	1	Additive
	AmB + Colistin	0.25	0.125	2000	2000	0.5	1	1.5	Indifference
	Caspo + TM8	2	0.031	15.6	7.8	0.016	0.5	0.52	P. Synergy
	Caspo + RK758	2	2	12	12	1	1	2	Indifference
	Caspo + Colistin	2	0.5	2000	31.25	0.25	0.016	0.27	Synergy
*M. guilliermondii* 001	Fluco + TM8	4	1	7.8	1.95	0.25	0.25	0.5	Synergy
	Fluco + RK758	4	0.5	12	3	0.125	0.25	0.375	Synergy
	Fluco + Colistin	4	4	2000	2000	1	1	2	Indifference
	AmB + TM8	0.125	0.03125	7.8	3.9	0.25	0.5	0.75	P. Synergy
	AmB + RK758	0.125	0.0625	12	6	0.5	0.5	1	Additive
	AmB + Colistin	0.125	0.0625	2000	2000	0.5	0.5	1	Additive
	Caspo + TM8	4	0.25	7.8	3.9	0.0625	0.5	0.56	P. Synergy
	Caspo + RK758	4	0.25	12	6	0.0625	0.5	0.56	P. Synergy
	Caspo + Colistin	4	1	2000	250	0.25	0.125	0.38	Synergy
*C. parapsilosis* 001	Fluco + TM8	1	0.016	7.8	3.9	0.016	0.5	0.52	P. Synergy
	Fluco + RK758	1	0.25	48	6	0.25	0.125	0.38	Synergy
	Fluco + Colistin	1	0.5	2000	250	0.5	0.125	0.63	P. Synergy
	AmB + TM8	0.25	0.0625	7.8	1.95	0.25	0.25	0.5	Synergy
	AmB + RK758	0.25	0.125	48	12	0.50	0.25	0.75	P. Synergy
	AmB + Colistin	0.25	0.0625	2000	500	0.25	0.25	0.5	Synergy
	Caspo + TM8	8	0.0039	7.8	7.8	0.0005	1	1	Indifference
	Caspo + RK758	8	4	48	12	0.5	0.25	0.75	P. Synergy
	Caspo + Colistin	8	0.125	2000	500	0.016	0.25	0.27	Synergy
*K. marxianus* 001	Fluco + TM8	1	0.25	7.8	3.9	0.25	0.5	0.75	P. Synergy
	Fluco + RK758	1	0.25	12	3	0.25	0.25	0.5	Synergy
	Fluco + Colistin	1	1	4000	4000	1	1	2	Indifference
	AmB + TM8	0.25	0.0625	7.8	1.95	0.25	0.25	0.5	Synergy
	AmB + RK758	0.25	0.125	12	3	0.5	0.25	0.75	P. Synergy
	AmB + Colistin	0.25	0.0625	4000	2000	0.25	0.5	0.75	P. Synergy
	Caspo + TM8	2	0.5	7.8	1.95	0.25	0.25	0.5	Synergy
	Caspo + RK758	2	0.25	12	1.5	0.125	0.125	0.25	Synergy
	Caspo + Colistin	2	1	4000	2000	0.5	0.5	1	P. Synergy
*C. auris* 04	Fluco + TM8	128	16	31.2	7.8	0.125	0.25	0.38	Synergy
	Fluco + RK758	128	32	12	3	0.25	0.25	0.5	Synergy
	Fluco + Colistin	128	64	4000	1000	0.5	0.25	0.75	P. Synergy
	AmB + TM8	4	1	31.2	7.8	0.25	0.5	0.5	Synergy
	AmB + RK758	4	0.25	12	12	0.0625	1	1.06	Indifference
	AmB + Colistin	4	4	4000	4000	1	1	2	Indifference
	Caspo + TM8	1	0.5	31.2	7.8	0.5	0.25	0.75	P. Synergy
	Caspo + RK758	1	0.25	12	6	0.25	0.5	0.75	P. Synergy
	Caspo + Colistin	1	0.5	4000	2000	0.5	0.5	1	Additive

Abbreviations: FIC, fractional inhibitory concentration index; Fluco, fluconazole; AmB, amphotericin B; Caspo, caspofungin; P. synergy, partial synergy.

**Table 6:** Fold-change in MIC of peptidomimetics in the presence of exogenous ergosterol for yeasts.

Yeasts	Fold-Change in MIC (Increment)			
	TM8		RK758	
	Ergosterol (200 µg/mL)	Ergosterol (400 µg/mL)	Ergosterol (200 µg/mL)	Ergosterol (400 µg/mL)
*C. albicans* 002	4	4	4	4
*P. kudriavzeviii* 001	8	8	4	4
C. *tropicalis* 001	8	4	4	4
*N*. *glabratus* 001	4	4	8	4
*M. guilliermondii* 001	8	8	4	4
*C. parapsilosis* 001	4	4	4	4
C. *auris* 04	4	4	4	4

## Data Availability

The original contributions presented in this study are included in the article/supplementary material. Further inquiries can be directed to the corresponding author.

## Supplementary Materials

The following supporting information can be downloaded at https://www.mdpi.com/article/10.3390/jof11050370/s1: Table S1. MIC of peptidomimetics in the presence of sorbitol against yeast cells.

## References

[1] Mayer F.L., Wilson D., Hube B. (2013). Candida albicans pathogenicity mechanisms. Virulence.

[2] Turner S.A., Butler G. (2014). The Candida pathogenic species complex. Cold Spring Harb. Perspect. Med..

[3] World Health Organization (2022). WHO Fungal Priority Pathogens List to Guide Research, Development and Public Health Action.

[4] Zeidler U., Bougnoux M.-E., Lupan A., Helynck O., Doyen A., Garcia Z., Sertour N., Clavaud C., Munier-Lehmann H., Saveanu C. (2013). Synergy of the antibiotic colistin with echinocandin antifungals in Candida species. J. Antimicrob. Chemother..

[5] Denning D.W. (2024). Global incidence and mortality of severe fungal disease. Lancet Infect. Dis..

[6] Seyoum E., Bitew A., Mihret A. (2020). Distribution of Candida albicans and non-albicans Candida species isolated in different clinical samples and their in vitro antifungal suscetibity profile in Ethiopia. BMC Infect. Dis..

[7] Malinovská Z., Čonková E., Váczi P. (2023). Biofilm formation in medically important Candida species. J. Fungi.

[8] Takashima M., Sugita T. (2022). Taxonomy of Pathogenic Yeasts Candida, Cryptococcus, Malassezia, and Trichosporon Current Status, Future Perspectives, and Proposal for Transfer of Six Candida Species to the Genus Nakaseomyces. Med. Mycol. J..

[9] Liu F., Hu Z.D., Zhao X.M., Zhao W.N., Feng Z.X., Yurkov A., Alwasel S., Boekhout T., Bensch K., Hui F.L. (2024). Phylogenomic analysis of the Candida auris- Candida haemuli clade and related taxa in the Metschnikowiaceae, and proposal of thirteen new genera, fifty-five new combinations and nine new species. Persoonia—Mol. Phylogeny Evol. Fungi.

[10] Parambath S., Dao A., Kim H.Y., Zawahir S., Alastruey Izquierdo A., Tacconelli E., Govender N., Oladele R., Colombo A., Sorrell T. (2024). Candida albicans—A systematic review to inform the World Health Organization Fungal Priority Pathogens List. Med. Mycol..

[11] Lass-Flörl C., Kanj S.S., Govender N.P., Thompson III G.R., Ostrosky-Zeichner L., Govrins M.A. (2024). Invasive candidiasis. Nat. Rev. Dis. Primers.

[12] Nguyen T.A., Kim H.Y., Stocker S., Kidd S., Alastruey-Izquierdo A., Dao A., Harrison T., Wahyuningsih R., Rickerts V., Perfect J. (2024). Pichia kudriavzevii (Candida krusei): A systematic review to inform the World Health Organisation priority list of fungal pathogens. Med. Mycol..

[13] Mishra S.K., Yasir M., Willcox M. (2023). Candida auris: An emerging antimicrobial-resistant organism with the highest level of concern. Lancet Microbe.

[14] Kean R., Ramage G. (2019). Combined antifungal resistance and biofilm tolerance: The global threat of Candida auris. mSphere.

[15] Askari F., Kaur R. (2024). Candida glabrata: A Tale of Stealth and Endurance. ACS Infect. Dis..

[16] Savini V., Catavitello C., Onofrillo D., Masciarelli G., Astolfi D., Balbinot A., Febbo F., D’Amario C., D’Antonio D. (2011). What do we know about Candida guilliermondii? A voyage throughout past and current literature about this emerging yeast. Mycoses.

[17] Pfaller M.A., Carvalhaes C.G., DeVries S., Rhomberg P.R., Castanheira M. (2022). Impact of COVID-19 on the antifungal susceptibility profiles of isolates collected in a global surveillance program that monitors invasive fungal infections. Med. Mycol..

[18] Pappas P.G., Lionakis M.S., Arendrup M.C., Ostrosky-Zeichner L., Kullberg B.J. (2018). Invasive candidiasis. Nat. Rev. Dis. Primers.

[19] Fang W., Wu J., Cheng M., Zhu X., Du M., Chen C., Liao W., Zhi K., Pan W. (2023). Diagnosis of invasive fungal infections: Challenges and recent developments. J. Biomed. Sci..

[20] Cornely O.A., Sprute R., Bassetti M., Chen S.C.A., Groll A.H., Kurzai O., Lass-Flörl C., Ostrosky-Zeichner L., Rautemaa-Richardson R., Revathi G. (2025). Global guideline for the diagnosis and management of candidiasis: An initiative of the ECMM in cooperation with ISHAM and ASM. Lancet Infect. Dis..

[21] Bhattacharya S., Sae-Tia S., Fries B.C. (2020). Candidiasis and mechanisms of antifungal resistance. Antibiotics.

[22] Mishra S.K., Akter T., Urmi U.L., Enninful G., Sara M., Shen J., Suresh D., Zheng L., Mekonen E.S., Rayamajhee B. (2025). Harnessing Non-Antibiotic Strategies to Counter Multidrug-Resistant Clinical Pathogens with Special Reference to Antimicrobial Peptides and Their Coatings. Antibiotics.

[23] Larsen C.E., Larsen C.J., Franzyk H., Regenberg B. (2015). Antifungal properties of peptidomimetics with an arginine-[β-(2, 5, 7-tri-tert-butylindol-3-yl) alanine]-arginine motif against Saccharomyces cerevisiae and Zygosaccharomyces bailii. FEMS Yeast Res..

[24] Kuppusamy R., Willcox M., Black D.S., Kumar N. (2019). Short cationic peptidomimetic antimicrobials. Antibiotics.

[25] Browne K., Kuppusamy R., Walsh W.R., Black D.S., Willcox M.D., Kumar N., Chen R. (2023). Antimicrobial Peptidomimetics Prevent the Development of Resistance against Gentamicin and Ciprofloxacin in Staphylococcus and Pseudomonas Bacteria. Int. J. Mol. Sci..

[26] Kataria A., Sharma R., Sharma S., Singh B., Kaur G., Yakubu C.M. (2021). Recent applications of bio-engineering principles to modulate the functionality of proteins in food systems. Trends Food Sci. Technol..

[27] Méndez-Samperio P. (2014). Peptidomimetics as a new generation of antimicrobial agents: Current progress. Infect. Drug Resist..

[28] Urmi U.L., Vijay A.K., Willcox M.D., Attard S., Enninful G., Kumar N., Islam S., Kuppusamy R. (2024). Exploring the efficacy of peptides and mimics against Influenza A Virus, Adenovirus, and murine norovirus. Int. J. Mol. Sci..

[29] Toepfer S., Keniya M.V., Lackner M., Monk B.C. (2024). Azole Combinations and Multi-Targeting Drugs That Synergistically Inhibit Candidozyma auris. J. Fungi.

[30] Bibi M., Murphy S., Benhamou R.I., Rosenberg A., Ulman A., Bicanic T., Fridman M., Berman J. (2021). Combining colistin and fluconazole synergistically increases fungal membrane permeability and antifungal cidality. ACS Infect. Dis..

[31] Nielsen J.E., Alford M.A., Yung D.B.Y., Molchanova N., Fortkort J.A., Lin J.S., Diamond G., Hancock R.E., Jenssen H.v., Pletzer D. (2022). Self-assembly of antimicrobial peptoids impacts their biological effects on ESKAPE bacterial pathogens. ACS Infect. Dis..

[32] Jenssen H. (2025). Self-assembling peptoid-based antimicrobial nanomaterials. Nanomedicine.

[33] Mishra S.K., Yasir M., Kuppusamy R., Wong E.H.H., Hui A., Sørensen K., Lin J.S., Jenssen H., Barron A.E., Willcox M. (2025). Antimicrobial activity of peptoids against Metallo-β-lactamase-producing Klebsiella pneumoniae, Acinetobacter baumannii, Pseudomonas aeruginosa, and other WHO priority pathogens, including Candida auris. J. Appl. Microbiol..

[34] Critchley I.A., Douglas L.J. (1987). Role of glycosides as epithelial cell receptors for Candida albicans. Microbiology.

[35] Casalini G., Giacomelli A., Antinori S. (2024). The WHO fungal priority pathogens list: A crucial reappraisal to review the prioritisation. Lancet Microbe.

[36] Willcox M., Webb B., Thakur A., Harty D. (1998). Interactions between Candida species and platelets. J. Med. Microbiol..

[37] Webb B., Willcox M., Thomas C., Harty D., Knox K. (1995). The effect of sodium hypochlorite on potential pathogenic traits of Candida albicans and other Candida species. Oral Microbiol. Immunol..

[38] Kurtzman C.P., Suzuki M. (2010). Phylogenetic analysis of ascomycete yeasts that form coenzyme Q-9 and the proposal of the new genera Babjeviella, Meyerozyma, Millerozyma, Priceomyces, and Scheffersomyces. Mycoscience.

[39] Chatterjee P., Choi H., Ochoa B., Garmon G., Coppin J.D., Allton Y., Lukey J., Williams M.D., Navarathna D., Jinadatha C. (2020). Clade-specific variation in susceptibility of Candida auris to broad-spectrum ultraviolet C light (UV-C). Infect. Control Hosp. Epidemiol..

[40] Kuppusamy R., Yasir M., Yu T.T., Voli F., Vittorio O., Miller M.J., Lewis P., Black D.S., Willcox M., Kumar N. (2023). Tuning the anthranilamide peptidomimetic design to selectively target planktonic bacteria and biofilm. Antibiotics.

[41] (2024). PubChem Compound Summary for CID 3365, Fluconazole. https://pubchem.ncbi.nlm.nih.gov/compound/Fluconazole.

[42] (2024). PubChem Compound Summary for CID 139586083. https://pubchem.ncbi.nlm.nih.gov/compound/Caspofungin.

[43] (2024). PubChem Compound Summary for CID 5280965, Amphotericin B. https://pubchem.ncbi.nlm.nih.gov/compound/Amphotericin-B.

[44] (2024). PubChem Compound Summary for CID 133109993. https://pubchem.ncbi.nlm.nih.gov/compound/Colistin.

[45] CLSI (2008). Reference Method for Broth Dilution Antifungal Susceptibility Testing of Yeasts.

[46] CLSI (2022). Epidemiological Cutoff Values for Antifungal Susceptibility Testing.

[47] Black C., Al Mahmud H., Howle V., Wilson S., Smith A.C., Wakeman C.A. (2023). Development of a polymicrobial checkerboard assay as a tool for determining combinatorial antibiotic effectiveness in polymicrobial communities. Antibiotics.

[48] Timurkaynak F., Can F., Azap Ö.K., Demirbilek M., Arslan H., Karaman S.Ö. (2006). In vitro activities of non-traditional antimicrobials alone or in combination against multidrug-resistant strains of Pseudomonas aeruginosa and Acinetobacter baumannii isolated from intensive care units. Int. J. Antimicrob. Agents.

[49] Mun S.-H., Joung D.-K., Kim Y.-S., Kang O.-H., Kim S.-B., Seo Y.-S., Kim Y.-C., Lee D.-S., Shin D.-W., Kweon K.-T. (2013). Synergistic antibacterial effect of curcumin against methicillin-resistant Staphylococcus aureus. Phytomedicine.

[50] Haji S.H., Ali F.A., Aka S.T.H. (2022). Synergistic antibacterial activity of silver nanoparticles biosynthesized by carbapenem-resistant Gram-negative bacilli. Sci. Rep..

[51] Pankey G., Ashcraft D., Kahn H., Ismail A. (2014). Time-kill assay and Etest evaluation for synergy with polymyxin B and fluconazole against Candida glabrata. Antimicrob. Agents Chemother..

[52] Escalante A., Gattuso M., Pérez P., Zacchino S. (2008). Evidence for the Mechanism of Action of the Antifungal Phytolaccoside B Isolated from Phytolacca tetramera Hauman. J. Nat. Prod..

[53] Frost D.J., Brandt K.D., Cugier D., Goldman R. (1995). A whole-cell Candida albicans assay for the detection of inhibitors towards fungal cell wall synthesis and assembly. J. Antibiot..

[54] Zorić N., Kosalec I., Tomić S., Bobnjarić I., Jug M., Vlainić T., Vlainić J. (2017). Membrane of Candida albicans as a target of berberine. BMC Complement. Altern. Med..

[55] Merlino F., Carotenuto A., Casciaro B., Martora F., Loffredo M.R., Di Grazia A., Yousif A.M., Brancaccio D., Palomba L., Novellino E. (2017). Glycine-replaced derivatives of [Pro3, DLeu9] TL, a temporin L analogue: Evaluation of antimicrobial, cytotoxic and hemolytic activities. Eur. J. Med. Chem..

[56] Kondori N., Baltzer L., Dolphin G., Mattsby-Baltzer I. (2011). Fungicidal activity of human lactoferrin-derived peptides based on the antimicrobial αβ region. Int. J. Antimicrob. Agents.

[57] Richardson M., Smith H. (1981). Production of germ tubes by virulent and attenuated strains of Candida albicans. J. Infect. Dis..

[58] CLSI (2022). Performance Standards for Antifungal Susceptibility Testing of Yeasts.

[59] CDC Antifungal Susceptibility Testing for *C. auris*. https://www.cdc.gov/candida-auris/hcp/laboratories/antifungal-susceptibility-testing.html.

[60] Harris M.R., Coote P.J. (2010). Combination of caspofungin or anidulafungin with antimicrobial peptides results in potent synergistic killing of Candida albicans and Candida glabrata in vitro. Int. J. Antimicrob. Agents.

[61] Lu H., Shrivastava M., Whiteway M., Jiang Y. (2021). Candida albicans targets that potentially synergize with fluconazole. Crit. Rev. Microbiol..

[62] Berkow E.L., Lockhart S.R. (2017). Fluconazole resistance in Candida species: A current perspective. Infect. Drug Resist..

[63] Katsipoulaki M., Stappers M.H.T., Malavia-Jones D., Brunke S., Hube B., Gow N.A.R. (2024). *Candida albicans* and *Candida glabrata*: Global priority pathogens. Microbiol. Mol. Biol. Rev..

[64] Lindberg E., Hammarström H., Ataollahy N., Kondori N. (2019). Species distribution and antifungal drug susceptibilities of yeasts isolated from the blood samples of patients with candidemia. Sci. Rep..

[65] Jamiu A.T., Albertyn J., Sebolai O.M., Pohl C.H. (2020). Update on Candida krusei, a potential multidrug-resistant pathogen. Med. Mycol..

[66] Ben-Ami R. (2018). Treatment of invasive candidiasis: A narrative review. J. Fungi.

[67] Pappas P.G., Kauffman C.A., Andes D.R., Clancy C.J., Marr K.A., Ostrosky-Zeichner L., Reboli A.C., Schuster M.G., Vazquez J.A., Walsh T.J. (2016). Clinical practice guideline for the management of candidiasis: 2016 update by the Infectious Diseases Society of America. Clin. Infect. Dis..

[68] Perlin D.S. (2015). Echinocandin resistance in Candida. Clin. Infect. Dis..

[69] Wang Y., Yan H., Li J., Zhang Y., Wang Z., Sun S. (2023). Antifungal activity and potential mechanism of action of caspofungin in combination with ribavirin against Candida albicans. Int. J. Antimicrob. Agents.

[70] Prayag P.S., Patwardhan S.A., Joshi R.S., Dhupad S., Rane T., Prayag A.P. (2024). Comparative efficacies of the three echinocandins for Candida auris candidemia: Real world evidence from a tertiary centre in India. Med. Mycol..

[71] Pierce C.G., Srinivasan A., Uppuluri P., Ramasubramanian A.K., López-Ribot J.L. (2013). Antifungal therapy with an emphasis on biofilms. Curr. Opin. Pharmacol..

[72] Ahmady L., Gothwal M., Mukkoli M.M., Bari V.K. (2024). Antifungal drug resistance in Candida: A special emphasis on amphotericin B. APMIS.

[73] Zhu P., Li Y., Guo T., Liu S., Tancer R.J., Hu C., Zhao C., Xue C., Liao G. (2023). New antifungal strategies: Drug combination and co-delivery. Adv. Drug Deliv. Rev..

[74] Shaban S., Patel M., Ahmad A. (2023). Fungicidal activity of human antimicrobial peptides and their synergistic interaction with common antifungals against multidrug-resistant Candida auris. Int. Microbiol..

[75] Balkrishna A., Kharayat B., Rastogi S., Kabdwal M., Haldar S., Varshney A. (2023). Withania somnifera seed oil exhibits antibiofilm properties against drug-resistant Candida auris clinical isolate through modulation in cell permeability. J. Appl. Microbiol..

[76] Park J., Kim H., Kang H.-K., Choi M.-C., Park Y. (2022). Lycosin-II exhibits antifungal activity and inhibits dual-species biofilm by Candida albicans and Staphylococcus aureus. J. Fungi.

[77] Kretschmar M., Hube B., Bertsch T., Sanglard D., Merker R., Schröder M., Hof H., Nichterlein T. (1999). Germ tubes and proteinase activity contribute to virulence of Candida albicans in murine peritonitis. Infect. Immun..

[78] Li X., Hu Q., Lin Q., Luo J., Xu J., Chen L., Xu L., Lin X. (2022). Inhibition of Candida albicans in vivo and in vitro by antimicrobial peptides chromogranin A-N12 through microRNA-155/suppressor of cytokine signaling 1 axis. Bioengineered.

[79] D’Auria F.D., Casciaro B., De Angelis M., Marcocci M.E., Palamara A.T., Nencioni L., Mangoni M.L. (2022). Antifungal activity of the frog skin peptide temporin G and its effect on Candida albicans virulence factors. Int. J. Mol. Sci..

[80] Cesare G.B.D., Cristy S.A., Garsin D.A., Lorenz M.C. (2020). Antimicrobial Peptides: A New Frontier in Antifungal Therapy. mBio.

[81] Sara M., Yasir M., Kalaiselvan P., Hui A., Kuppusamy R., Kumar N., Chakraborty S., Yu T.T., Wong E.H.H., Molchanova N. (2024). The activity of antimicrobial peptoids against multidrug-resistant ocular pathogens. Cont. Lens Anterior Eye.

[82] Bidaud A., Djenontin E., Botterel F., Chowdhary A., Dannaoui E. (2020). Colistin interacts synergistically with echinocandins against Candida auris. Int. J. Antimicrob. Agents.

[83] Yousfi H., Ranque S., Rolain J.-M., Bittar F. (2019). In vitro polymyxin activity against clinical multidrug-resistant fungi. Antimicrob. Resist. Infect. Control..

[84] Schwarz P., Nikolskiy I., Bidaud A.-L., Sommer F., Bange G., Dannaoui E. (2022). In vitro activity of amphotericin B in combination with colistin against fungi responsible for invasive infections. J. Fungi.

[85] Carton J.D., de-la-Fuente I., Sevillano E., Jauregizar N., Quindós G., Eraso E., Guridi A. (2025). In Vitro Assessment of Fluconazole and Cyclosporine A Antifungal Activities: A Promising Drug Combination Against Different Candida Species. J. Fungi.

[86] Cowen L.E., Singh S.D., Köhler J.R., Collins C., Zaas A.K., Schell W.A., Aziz H., Mylonakis E., Perfect J.R., Whitesell L. (2009). Harnessing Hsp90 function as a powerful, broadly effective therapeutic strategy for fungal infectious disease. Proc. Natl. Acad. Sci. USA.

