# Supplementary material for: Evaluation of the Synergistic Activity of Antimicrobial Peptidomimetics or Colistin Sulphate with Conventional Antifungals Against Yeasts of Medical Importance
Source: J Fungi (Basel). 2025 May 12;11(5):370. doi: 10.3390/jof11050370 (PMC12112644; doi:10.3390/jof11050370)
Supplement: Supplementary file 1 [file jof-11-00370-s001.zip › jof-3595328-supplementary.pdf]

Table S1. MIC of peptidomimetics in the presence of sorbitol against yeast cells

Yeasts	MIC							
	TM8 (µg/mL)				RK758 (µg/mL)			
	2 days		7 days		2 days		7 days	
	Sorbitol (-)	Sorbitol (0.8 M)	Sorbitol (-)	Sorbitol (0.8 M)	Sorbitol (-)	Sorbitol (0.8 M)	Sorbitol (-)	Sorbitol (0.8 M)
C. albicans 002	15.6	15.6	15.6	15.6	12	12	12	12
P. kudriavzeviii 001	7.8	7.8	7.8	7.8	12	12	12	12
C. tropicalis 001	7.8	7.8	7.8	7.8	12	12	12	12
N. glabratus 001	15.6	15.6	15.6	15.6	12	12	12	12
M. guilliermondii 001	7.8	7.8	7.8	7.8	12	12	12	12
C. parapsilosis 001	7.8	7.8	7.8	7.8	48	48	48	48
C. auris 0384	31.2	31.2	31.2	31.2	12	12	12	12
